# Highly transparent and flexible polyaniline mesh sensor for chemiresistive sensing of ammonia gas†

**DOI:** 10.1039/c7ra13516e

**Published:** 2018-01-30

**Authors:** Jingxuan Cai, Cuiping Zhang, Arshad Khan, Chuwei Liang, Wen-Di Li

**Affiliations:** Department of Mechanical Engineering, The University of Hong Kong Pokfulam Hong Kong China; HKU-Zhejiang Institute of Research and Innovation (HKU-ZIRI) Hangzhou 311300 Zhejiang China liwd@hku.hk

## Abstract

A new structure of a flexible, transparent polyaniline (PANI) ammonia gas sensor is reported. The sensor features a hierarchical nanostructured PANI polymer arranged in a micromesh, exhibiting excellent chemiresistive sensitivity to ammonia gas and near-neutral color transparency. The PANI mesh is embedded in a flexible substrate and therefore exhibits superior mechanical stability against peeling and bending. These merits make it a promising candidate for application in wearable electronics. Moreover, the PANI mesh sensor is fabricated through a cost-effective, solution-based strategy that enables vacuum-free fabrication of a sacrificial catalytic copper mesh followed by *in situ* polymerization, and this strategy is scalable for high-volume production. We demonstrate the high-performance resistive sensing of ammonia gas with concentrations from 2.5 ppb to 100 ppm using this flexible PANI mesh sensor with an excellent transparency of 88.4% at 600 nm wavelength. Furthermore, no significant degradation in the sensing performance occurs after 1000 bending cycles.

## Abstract 2

A new solution to flexible transparent polyaniline sensors by introducing hierarchical structures with regular micro meshes and solution-processed fabrication.

## Graphical abstract

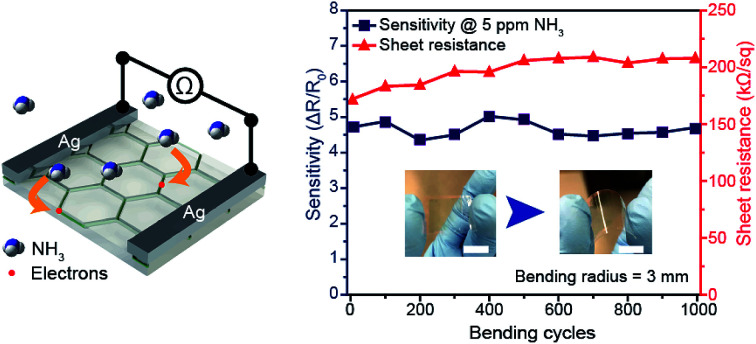

## Introduction

Recently, due to the increasing demand for portable health monitoring applications, wearable electronic devices, such as electronic noses,^1^ smart skin,^2^ and smart wristbands,^3^ have been extensively studied. High-performance compact bio- and gas sensors are key for these applications, and efforts have been devoted to developing sensors meeting these demands. For example, a nanoplasmonic immunosensor has been constructed on few-layers MoS_2_ for cytokine optoelectronic immunosensing.^4^ Graphene nanoribbon arrays for NO_2_ gas sensing,^5^ silver nanowires-embedded polydimethylsiloxane electrode for pressure sensing,^6^ silicon multi-nanochannel FETs for detection of insulin in serum,^7^ and fluorescence resonance energy transfer based sensors utilizing conjugated polyelectrolyte for DNA sensing,^8^ have also been investigated. To fulfill the requirements of portable and real-time detection of trace amounts of hazardous gases in wearable health monitoring devices, high-performance thin-film sensors with excellent flexibility and transparency to be integrated in wearable optoelectronic devices are desirable. With a transparent appearance, the developed sensor can be better integrated into other devices with minimal interference of their optoelectronic functions. For example, ammonia is a volatile chemical that is widely used in various industries, and is a corrosive, poisonous and highly toxic gas. Exposure to ammonia may cause severe harm to the skin, eyes or respiratory tract, and remaining in an environment with a high concentration of ammonia may even cause dyspnea or death.^9^ Thus, a flexible, highly sensitive sensor that is capable of detecting trace amounts of ammonia is desirable as an integrated wearable personal protection device during the safe handling of ammonia gas.

Most commonly used gas sensors are usually made from metal oxide semiconductors, which are often operated at elevated temperatures (above 200 °C) and lack flexibility or transparency.^10,11^ The development of different gas sensors based on nanostructured materials for the efficient and rapid detection of trace harmful gases has recently become a fast-growing field. In particular, electrically conductive nanostructured polymers have been suggested as promising candidates for chemiresistive gas sensors due to the strong response of their conductivity to specific gases.^12–14^ Among the conductive polymers, polyaniline (PANI), which is operated at room temperature and is pH-sensitive, has been extensively studied because of its simple synthesis, high sensitivity, good flexibility and excellent reliability.^15^ Fabrication of flexible, transparent ammonia gas sensors based on PANI has recently been reported. For example, hierarchical nanostructured PANI networks have been fabricated on a polyethylene terephthalate (PET) film using a sacrificial silver nanowire template.^16^ The hierarchical nanostructured PANI networks exhibited 65% transmittance at 550 nm and a sensitivity of 9, which is defined as the relative change in the electrical resistance of ammonia gas sensors, under exposure to ammonia gas of 10 ppm. Hybrid structures combining PANI with graphene,^17^ carbon nanotubes,^18^ and reduced graphene oxide^19^ as well as inorganic materials such as zinc oxide^20^ and titanium oxide^21^ have also been investigated. However, in many existing works, the procedure for fabricating the sensor is still complicated and expensive, the optical transmittance of the sensors is not satisfactory, and the reproducibility remains a challenge. Currently, most PANI sensors are constructed through catalyst-free routes that produce PANI with low yields. A limited number of reports are available on the use of inorganic Cu(ii) salts^22^ and a *cis*-bis(glycinato)copper(ii) complex^23^ for the synthesis of PANI. However, the random distribution of the catalyst often leads to overoxidation to the pernigraniline form of PANI and the generation of various other by-products. These limitations call for improved and scalable fabrication process and new device structures for gas sensors that are desirable for wearable environmental monitoring devices.

Herein, we report the templated fabrication of a transparent chemiresistive sensing film of a hierarchical nanostructured PANI micromesh *via in situ* polymerization of aniline on a sacrificial Cu micromesh template embedded in a cyclic olefin copolymer (COC) substrate. The template Cu micromesh on COC is fabricated by photolithography, electrodeposition and subsequent thermal imprint transfer. The catalytic Cu micromesh not only provides a guided template for the selective deposition of nanostructured PANI but also enhances the deposition yield of the PANI, thus enhancing the transparency of the gas sensor without sacrificing its sensing performance. The assembled sensor exhibits high-performance sensing of ammonia gas with concentrations ranging from 2.5 ppb to 100 ppm and excellent near-neutral color transparency (88.4% at 600 nm). The embedded structure also improves the mechanical stability of the sensor under peeling and high bending stress, with no significant decrease in the sensing performance observed after 1000 bending cycles.

## Experimental

### Material

Aniline was purchased from J&K Chemical (Hong Kong, China) and used without further purification. Ammonium peroxodisulfate (APS) and perfluorodecyltrichlorosilane (FDTS) were obtained from Alfa Aesar (Lancashire, United Kingdom). Hydrochloric acid was obtained from VWR (Batavia, USA). The COC film (Grade 8007) was commercially available from TOPAS Advanced Polymer (Frankfurt, Germany). Indium tin oxide (ITO) glass was obtained from South China Xiang Science & Technology (Shenzhen, China). Cu sulfate pentahydrate, sulfuric acid, and sodium dodecyl sulfate were purchased from Acros Organics (Geel, Belgium). Ethanol, 2-propanol, acetone, acetic acid, formaldehyde, toluene, and sodium bicarbonate were purchased from J&K Chemical (Hong Kong, China).

### Preparation of Cu mesh

The preparation method for the Cu mesh was modified from our previously published method. A hexagonal pattern was first fabricated on a 1.8 μm-thick AZ1500 photoresist-coated ITO glass substrate (3 × 3 cm^2^) after exposure to a 365 nm UV source (55.5 mJ cm^−2^) through a photomask. A 1.5 μm-thick hexagonal Cu mesh was then electrodeposited in the trench of the photoresist-coated ITO glass in a Cu electrolyte (Caswell, New York, USA) with a current density of 5 mA cm^−2^. After removal of the photoresist with acetone, the Cu mesh was transferred to a COC film by hot embossing.

### Preparation of PANI mesh

The Cu mesh-embedded COC film was first treated by oxygen plasma for 30 seconds, and an FDTS self-assembled monolayer coating was then prepared on the film by a vapor phase deposition process operated at 95 °C for 15 min. The PANI mesh on the COC film was fabricated *via in situ* chemical oxidative polymerization of aniline. In a typical procedure, the aniline monomer (2.4 mmol) was added into a precooled 1 M hydrochloric acid aqueous solution (20 mL) in a beaker and dispersed using ultrasonic vibration at 0.5 °C. Then, 20 mL precooled aqueous solution of 1 M hydrochloric acid and 1 M APS was poured into the aniline monomer solution, and the mixture was shaken for 30 seconds. Afterward, the Cu mesh-embedded COC film was immersed in the solution for the etching of Cu and the polymerization and deposition of aniline with the existence of oxidizing APS. The temperature of the polymerization process was maintained at approximately 0.5 °C by an ice bath. After immersion for various lengths of time, the polymerized COC films were thoroughly rinsed with DI water and ethanol, dried under a flow of nitrogen and baked in an oven at 70 °C for 30 min to remove absorbed water.

### Gas sensing measurements

The gas sensing measurements were performed on a WS-30A sensing system (Zhengzhou Winsen Electronics Technology, China) equipped with an 18 L chamber, a hot plate and two fans for mixing the gas. The relative humidity and temperature were maintained at approximately 40% and 24 °C during the experiments. The PANI mesh-embedded COC films were cut to 1.5 × 1.5 cm^2^ and attached to probes installed in the sensing system using silver paste. Calculated amounts of ammonium hydroxide (25 wt% NH_3_ in H_2_O) were dropped onto the hot plate in the chamber by a pipette to rapidly generate 5 ppm, 10 ppm, 50 ppm, and 100 ppm ammonia gas in the chamber, and the resistance of the film during exposure to a certain concentration of ammonia gas was recorded by the sensing system. To generate 100 ppb, 200 ppb, 500 ppb, and 1000 ppb ammonia gas, 1 wt% ammonia hydroxide was used. To generate 2.5 ppb, 5 ppb, 10 ppb, and 25 ppb ammonia gas, 0.1 wt% ammonia hydroxide was used. Thereafter, the PANI mesh was recovered under exposure to air by removing the chamber. To examine the gas sensing selectivity of the PANI mesh, other gas species, including ethanol, 2-propanol, acetic acid, acetone, formaldehyde, toluene, and carbon dioxide (generated from sodium bicarbonate) were tested by the same method.

### General characterization

The morphology of the samples was characterized using an S-4800N SEM (Hitachi, Japan) and an LEO 1530 Gemini SEM (Zeiss, Germany), and a Multimode-8 atomic force microscope (Bruker, USA). UV-vis transmittance spectra were collected on an HR2000+ ultraviolet/visible/near-infrared spectrometer (Ocean Optics, USA). All transmittance values presented in this paper were normalized to the absolute transmittance through the bare COC film substrate. Raman spectra were collected on a QE65 Pro Raman spectrometer (Ocean Optics, USA) using a 785 nm laser source. X-ray diffraction (XRD) spectra were recorded on a SmartLab X-ray diffractometer (Rigaku, Japan). The sheet resistance was measured by a Keithley 2400 source meter with a four-probe tester.

## Results and discussion

**Fig. 1:**
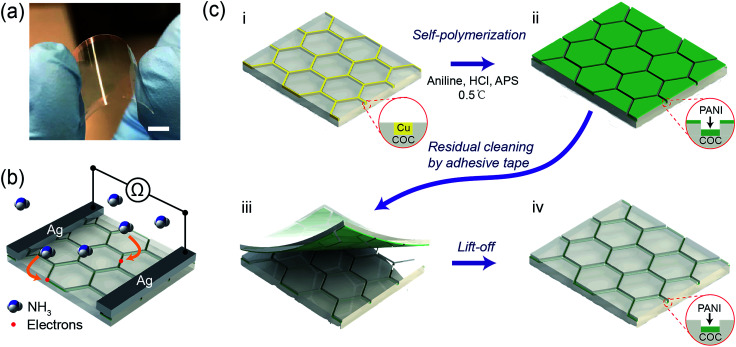
Schematic illustration of the fabrication of a hexagonal hierarchical PANI micromesh *via in situ* polymerization of aniline on a sacrificial Cu micromesh template. (a) Photograph of the transparent PANI mesh ammonia gas sensor. (b) Sensing mechanism of the PANI ammonia sensor. (c) (i) The sacrificial Cu mesh template; (ii) deposition of the hierarchical PANI micromesh on the Cu template *via in situ* oxidative polymerization of aniline; (iii) removal of the excess residual PANI molecules from the exposed COC by adhesive tape; and (iv) a hexagonal hierarchical PANI micromesh embedded in the COC film. The scale bar represents 1 cm.

Typically, COC films functionalized with a hierarchical PANI micromesh were fabricated through *in situ* polymerization of aniline on a Cu mesh-embedded COC film (Fig. 1). Fig. 1a displays a photo of a transparent PANI ammonia gas sensor prototype. The sensing mechanism of the PANI ammonia sensor is schematically illustrated in Fig. 1b. Upon exposure to a certain concentration of ammonia gas, ammonia molecules chemically bind to the protonated PANI, thus decreasing the conductivity of the PANI polymer by a reversible transition from the emeraldine salt form of PANI to its emeraldine base.^24^ Hence, by measuring the resistance across two silver electrodes on the transparent PANI gas sensor during exposure to ammonia gas, the concentration of the environmental ammonia gas can be determined.

The initial template Cu micromesh with a high transparency was fabricated on a 100 μm-thick COC (Grade 8007) film through a strategy consisting of photolithography, electrodeposition and an imprint transfer process, which has been thoroughly discussed in our previous report.^25,26^ The transparent electrode provides numerous merits, such as high conductivity and excellent flexibility, and a full-plastic bifacial dye-sensitized solar cell has been constructed on Ni-mesh electrode.^27^ The sheet resistance and transparency of the Cu mesh were 0.3 Ω sq^−1^ and 72%, respectively (Fig. S1, ESI†). The hexagonal Cu micromesh was embedded in the COC film and could be etched by ammonium persulfate (APS). Thus, the embedded Cu mesh could be employed as a sacrificial template for the *in situ* fabrication of flexible, highly transparent PANI mesh with excellent stability against mechanical deformation. Before the chemical polymerization process, the Cu mesh was treated with oxygen plasma for 30 seconds and coated with a self-assembled monolayer of perfluorodecyltrichlorosilane (FDTS) through vapor phase deposition to limit the deposition of PANI on the exposed surface of the COC film. Afterward, the Cu micromesh on COC was immersed in an aniline polymerization solution with an aniline concentration of 0.06 M and a molar ratio of [aniline] : [APS] = 1.2 : 1 for various lengths of time (Fig. 1c(i)). The protonated hierarchical PANI mesh was then deposited onto the Cu sacrificial template (Fig. 1c(ii)). Finally, the PANI film that was deposited on the exposed COC film was removed with an adhesive tape (Fig. 1c(iii)) to complete the fabrication of the PANI mesh embedded in the COC film as an ammonia gas sensor (Fig. 1c(iv)).

**Fig. 2:**
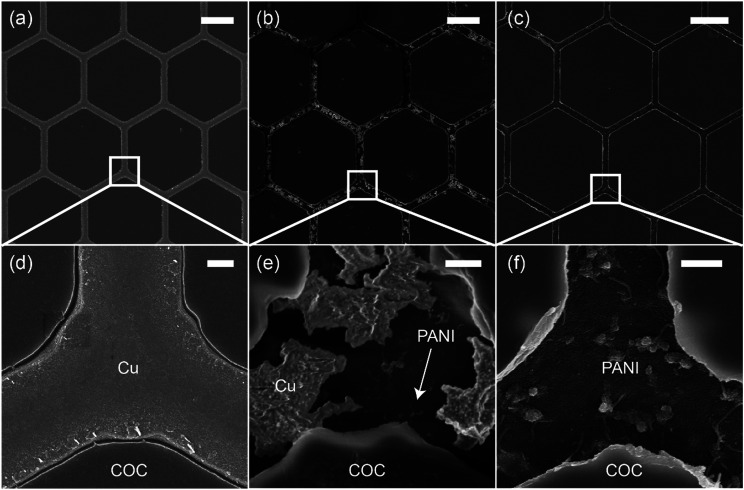
SEM images of (a) the original hexagonal Cu mesh and the PANI mesh after (b) 3 min and (c) 20 min in the aniline polymerization solution, respectively. (d)–(f) Magnified images of the trench junction area in (a)–(c). The scale bars in (a)–(c) and (d)–(f) represent 20 μm and 1 μm, respectively.

Fig. 2 shows scanning electron microscopy (SEM) images of the Cu mesh (Fig. 2a and d) and the PANI mesh (Fig. 2b, c, e and f) at different polymerization stages. The hexagonal Cu micromesh was fully embedded in the COC film and showed a smooth structure, resulting in a high conductivity and excellent stability. The standard reduction potential of Cu (+0.34 V) is lower than that of aniline (+1.02 V); thus, Cu is more likely than aniline to be oxidized by APS and forms Cu ions. As displayed in Fig. 2b and e, the Cu mesh was partially etched after immersion in the aniline polymerization solution for 3 min. The etching process of Cu was also confirmed by energy-dispersive X-ray spectroscopy (EDS) analysis, Cu was etched away in the oxidizing APS solution and complex [CuCl_4_]^2−^ was formed simultaneously (Fig. S2, ESI†). For a longer immersion time of 20 min, the Cu micromesh was completely etched away, and a hierarchical PANI micromesh consisting of PANI nanoparticles and nanofibers was deposited in the trenches containing the original sacrificial Cu template (Fig. 2c, f, and S3, ESI†). EDS analysis revealed that trace of Cu was observed in the deposited PANI mesh, which confirmed the existence of oxidized Cu after the polymerization process (Fig. S4, ESI†). A polymerization time of 20 min produced a PANI mesh with a thickness of approximately 100 nm (as measured by the atomic force microscope), and the average size of the deposited PANI nanoparticles was approximately 20 nm (Fig. S5, ESI†). Most of the PANI structures were deposited in the trenches rather than on the FDTS-treated COC surface because of the presence of catalytic Cu and Cu ions and the lower surface energy on COC surface. For comparison, an unmodified COC film was immersed in the aniline polymerization solution for 20 min, forming a PANI film with less particles and smoother surface, as shown in Fig. S6 (ESI†).

**Fig. 3:**
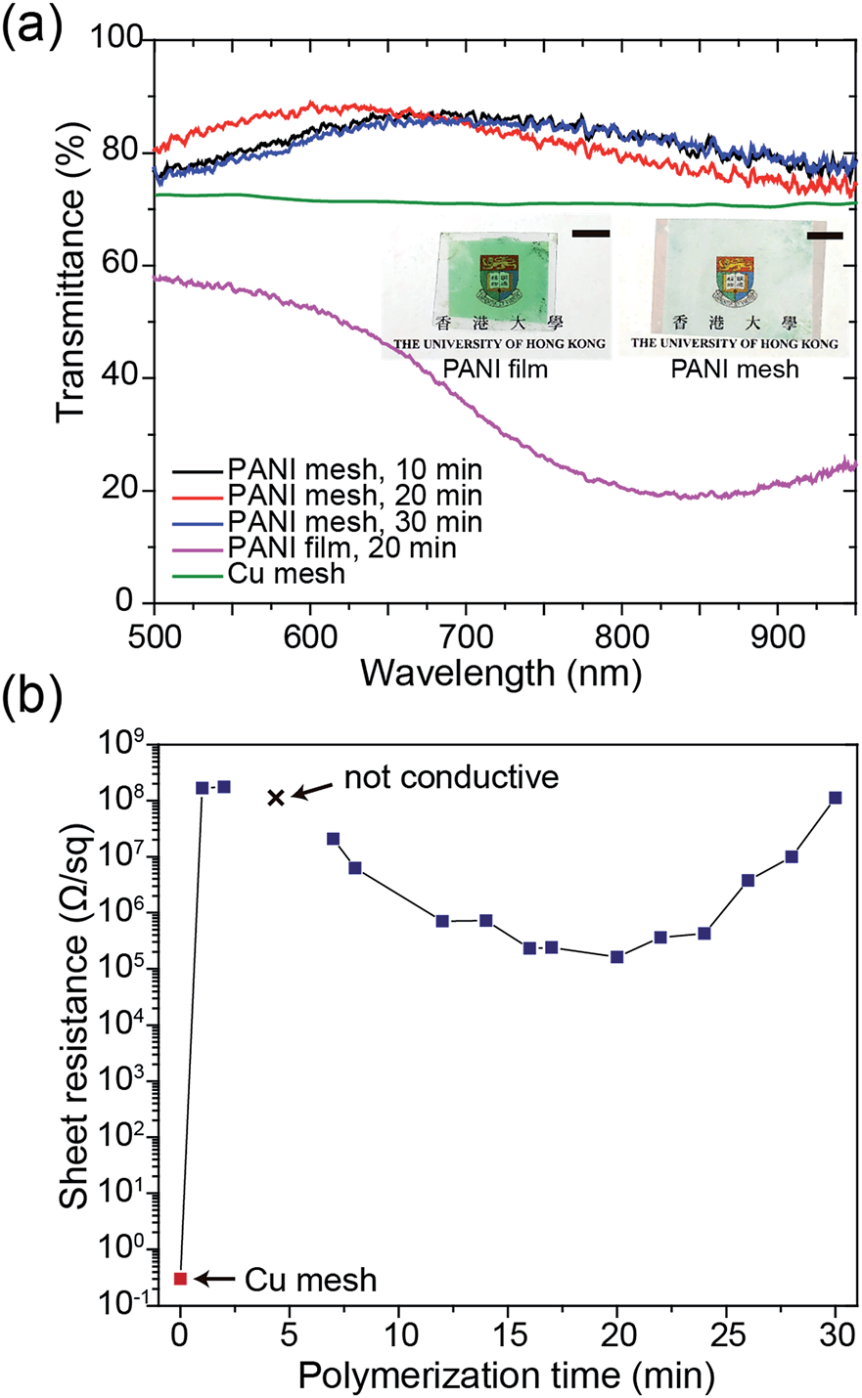
Optical and electrical characterization. (a) Transmittance spectra of the initial Cu mesh template, PANI film and PANI meshes after 10 min, 20 min, and 30 min in the aniline polymerization solution, respectively. (inset) Photographs of the PANI film (left) and the PANI micromesh (right) after 20 min in the aniline polymerization solution; the scale bars in the photograph represent 1 cm. (b) Sheet resistance of the original Cu mesh and the PANI meshes after immersion in the aniline polymerization solution for various lengths of time ranging from 1 min to 30 min.

The transparency and conductivity of the as-prepared PANI mesh changed with polymerization time because of the various amount of deposited PANI nanostructures. The optical transmittance of the PANI mesh after 10 min, 20 min, and 30 min in the aniline polymerization solution are illustrated in Fig. 3a. The nanostructured PANI mesh showed an excellent optical transparency across the range of 500 nm to 900 nm with a maximum transmittance of 88.4%, and this transparency was higher than that of the original Cu mesh (72%) because of the semi-transparent nature of PANI. Moreover, as displayed in the insets of Fig. 3a, the assembled gas sensor exhibited a near-neutral color. For comparison, the PANI film after immersion in the aniline polymerization solution for 20 min exhibited a transmittance of only 56.3%, and the film appeared green.

The sheet resistance of the PANI mesh changed with the time length of the oxidative polymerization and deposition of PANI on the COC substrate. As demonstrated in Fig. 3b, the sheet resistance of the original Cu mesh was 0.3 Ω sq^−1^, and after immersion in the aniline polymerization solution for 1 min, the sheet resistance increased to 167.0 MΩ sq^−1^ due to the oxidation and etching of the Cu by APS. After immersion in the aniline polymerization solution for 3 min, the film became non-conductive, as most of the Cu was etched away, and the amount of protonated PANI deposited in its place was not adequate to enable measurable conductivity. The conductivity of the PANI mesh then increased with increasing polymerization time up to 20 min, at which point the sheet resistance reached the minimum value of 161.6 kΩ sq^−1^. Then, the sheet resistance increased with increasing polymerization time above 20 min, which could be attributed to the further oxidation of the synthesized PANI from the emeraldine form to the non-conductive pernigraniline form in the presence of the catalytic Cu ions.^28^ This transformation was confirmed by the change in the appearance of the PANI film from green (emeraldine) to blue-green (mixture of emeraldine and pernigraniline), as displayed in Fig. S7 of the ESI.†

**Fig. 4:**
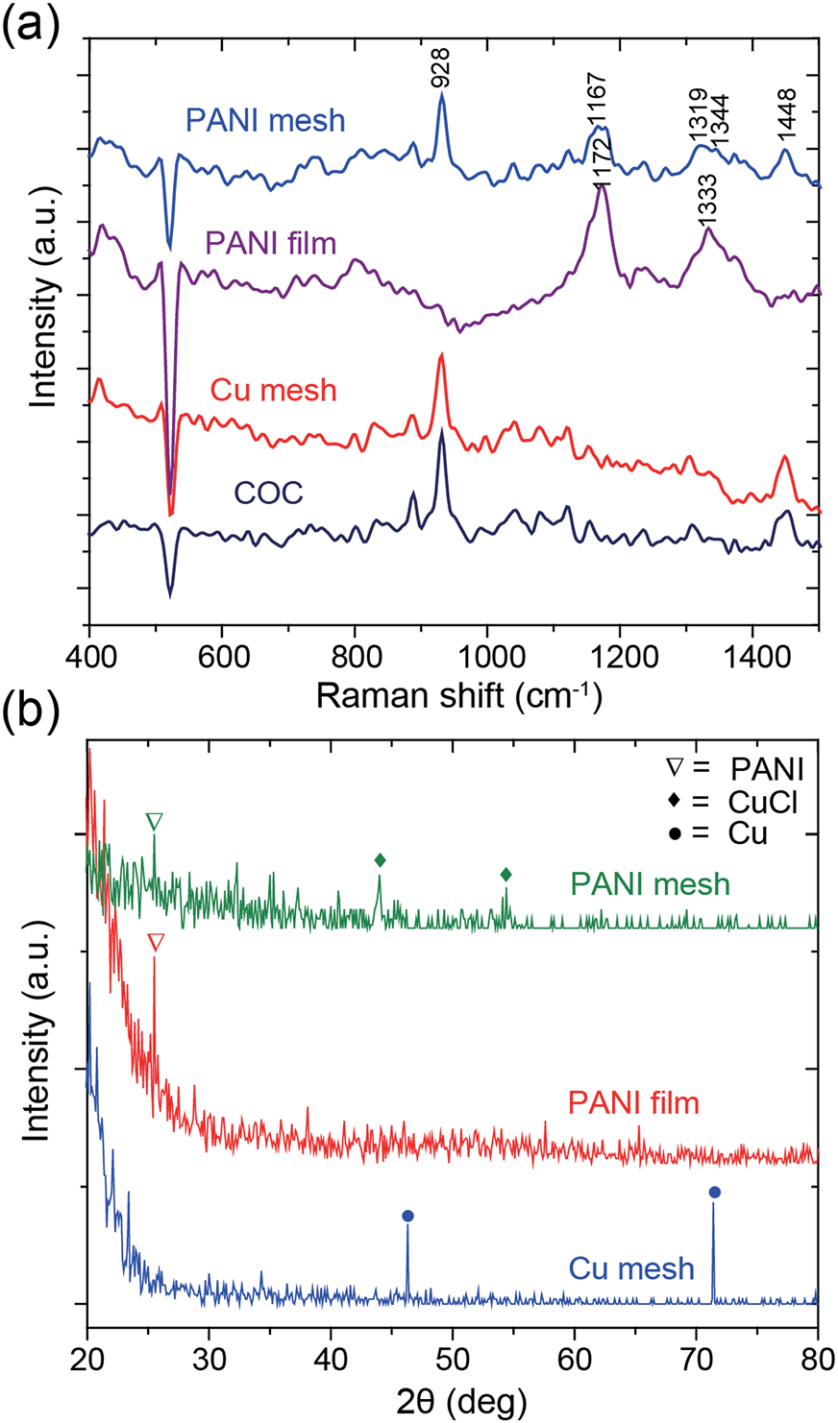
(a) Raman and (b) XRD spectra of the COC film, Cu mesh, PANI-coated COC film (PANI film) and PANI mesh. The aniline polymerization times for the PANI film and mesh were both 20 min.

To confirm the formation of a hierarchical nanostructured PANI mesh, the samples were characterized using Raman spectroscopy and X-ray diffraction (XRD). Fig. 4a presents the Raman spectra of the COC film, Cu mesh, PANI-coated COC film (PANI film) and PANI mesh. The aniline polymerization times for the PANI film and the PANI mesh were both 20 min. For the bare COC film, the peak at 928 cm^−1^ was attributed to the norbornene units of COC, and the weaker peak at 1448 cm^−1^ was assigned to the CH_2_ bending mode.^29^ On the PANI film, only peaks at 1172 and 1333 cm^−1^ were observed, which can be attributed to in-plane C–H bending and C–N*^+^ stretching vibrations in PANI, respectively, because the signal of the underlying COC film was masked by the PANI film. The peaks observed at 928, 1167, 1319, 1344, and 1448 cm^−1^ in the Raman spectrum obtained from the PANI mesh reveal the presence of PANI structures on the COC film.^30^ The shift in the peaks observed for the PANI mesh relative to the PANI film might be attributed to the interaction of PANI with Cu(ii) chloride and its complex, which was produced by the oxidization of Cu by APS in the presence of hydrochloric acid.

The PANI polymerization process was further analyzed by XRD spectroscopy to determine the existence of etched Cu. The spectral information of the samples is summarized in Fig. 4b. The peaks at 46.3° and 71.4° in the XRD spectrum of Cu mesh are attributed to the (111) and (220) planes of Cu, respectively.^31^ For the PANI film and the PANI mesh samples, the characteristic peak of PANI was observed at 25.4°, which is attributed to crystalline PANI. The observation of peaks at 44° and 54.4°, which are attributed to oxidized Cu(ii) chloride (CuCl) and its complex, and the absence of Cu peaks in the spectrum of the PANI mesh confirmed the transformation.^32^ The oxidized Cu(ii) also plays a key role in the catalytical deposition of the emeraldine form of PANI, as confirmed by a previous study.^33^ As displayed in Fig. S3 and S5 in the ESI,† Cu(ii) led to the formation of a dense and hierarchical nanostructured morphology of PANI.

**Fig. 5:**
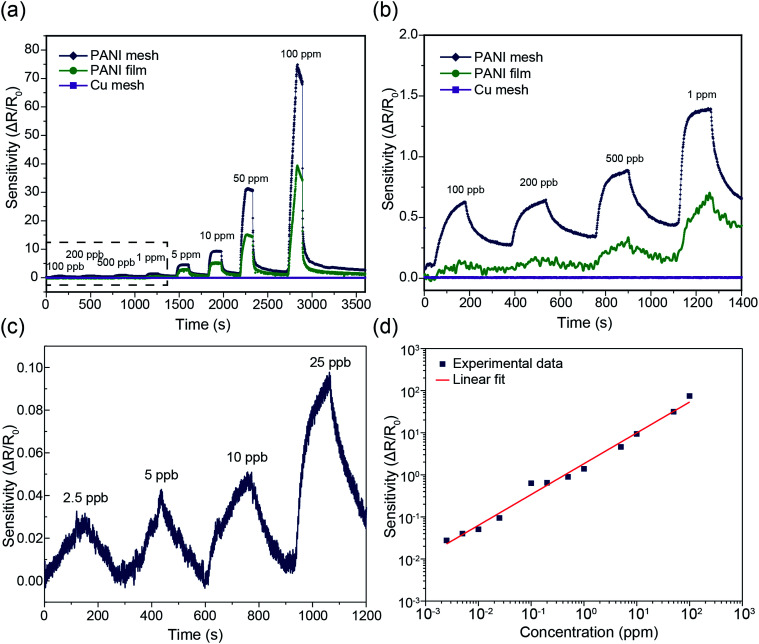
(a) Sensing performance of the Cu mesh, PANI mesh and PANI film with different concentrations of ammonia gas ranging from 100 ppb to 100 ppm. The PANI mesh and PANI film were generated from 20 min aniline polymerization. (b) Enlarged plot showing the sensing performance of the Cu mesh, PANI film and PANI mesh with different concentrations of ammonia gas ranging from 100 ppb to 1000 ppb (the dashed box in (a)). (c) Sensing performance of the PANI mesh with different concentrations of ammonia gas ranging from 2.5 ppb to 25 ppb. (d) Sensing sensitivity as a function of the ammonia gas concentration from 2.5 ppb to 100 ppm.

The hierarchical nanostructured PANI mesh is a promising candidate for a high-performance ammonia gas sensor with high transparency and excellent flexibility. The sensing performance of the PANI mesh, PANI film, and Cu mesh for ammonia gas were experimentally investigated. The sensing sensitivity was defined as the normalized change in resistance, *S* = (*R*_g_ − *R*_0_)/*R*_0_, where *R*_g_ is the resistance of the film after exposure to ammonia gas, and *R*_0_ is the resistance of the film in air. Fig. 5a displays the real-time variation in the resistance of the Cu micromesh, hierarchical nanostructured PANI micromesh, and PANI film polymerized for 20 min upon exposure to different concentrations of ammonia gas ranging from 100 ppb to 100 ppm. The response of the PANI mesh gradually increased with increasing concentration of ammonia and was higher than those of the PANI film, indicating that the small grain size increased the surface roughness of the hierarchical nanostructured PANI mesh. The increased roughness of the mesh resulted in a higher specific surface area, which led to a higher response upon exposure to the same concentration of ammonia as well as an enhanced signal-to-noise ratio (SNR) (Fig. S3 and S5, ESI†). In contrast, the PANI film exhibited only half of the sensitivity to the same concentration of ammonia gas, which is attributed to the smoother surface on the PANI film, as well as the increased deposition of emeraldine form of PANI with the presence of catalytic Cu ions for the PANI mesh^33^ (Fig. S6, ESI†). The detection limit of the PANI mesh for ammonia gas was as low as 2.5 ppb (Fig. 5c), which makes the PANI mesh a superior candidate for the real-time, low-concentration detection of ammonia gas. Fig. 5d demonstrates the variation in the sensing response as a function of the ammonia gas concentration; the curve implies that relationship between the response of our PANI mesh and the concentration of ammonia gas was highly linear (coefficient of determination *r*^2^ = 0.988). Compared with other ammonia gas sensors constructed on hierarchical nanostructured PANI, our PANI sensor prototype showed an improved transparency with a comparable sensitivity.^15,16,34^ Note that although a graphene–polyaniline nanocomposite film showed a slightly higher transparency (92.5% at a 550 nm wavelength),^35^ our PANI ammonia sensor prototype has a near-neutral color, a higher sensitivity (75 at 100 ppm ammonia gas), and a simplified fabrication approach.

**Fig. 6:**
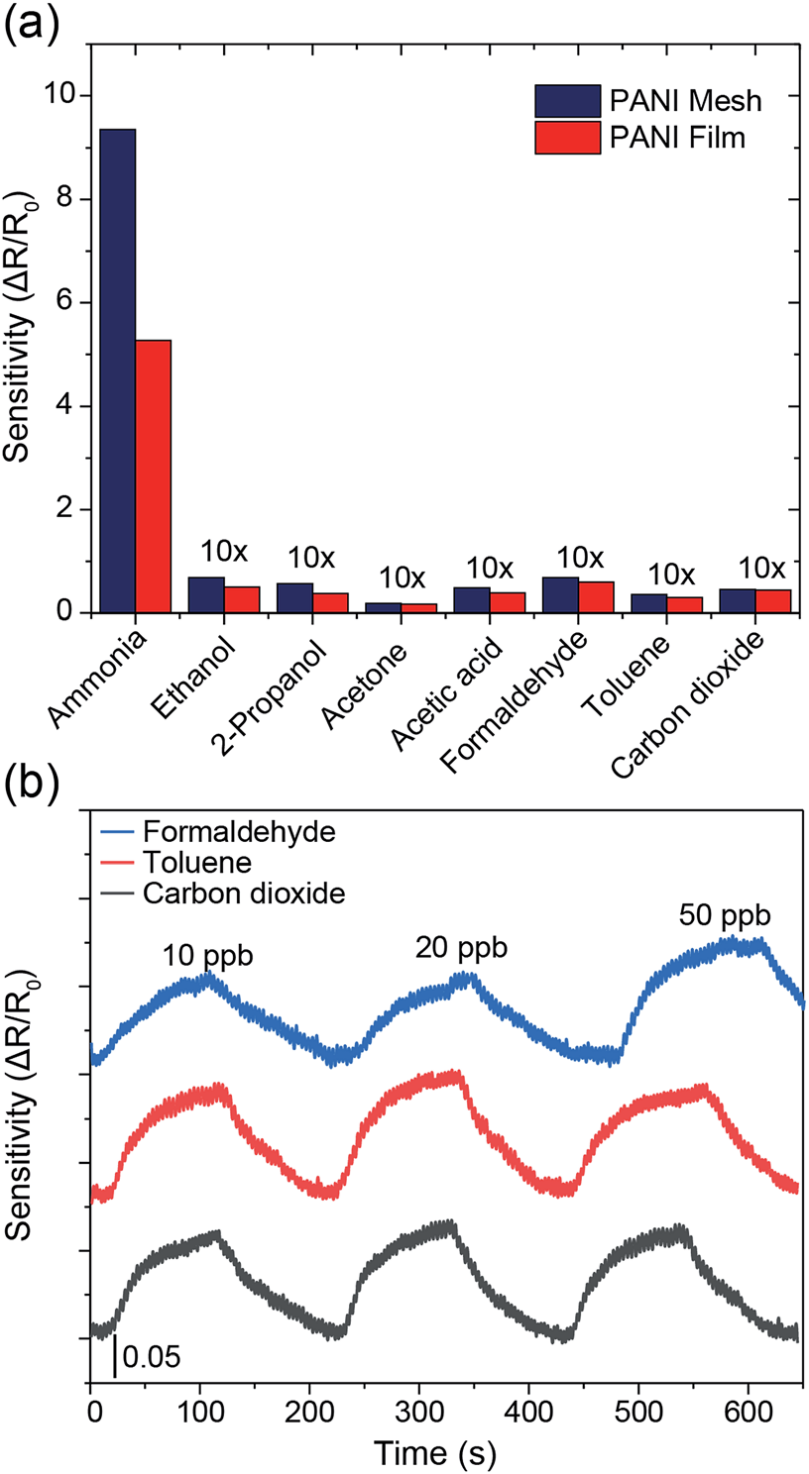
(a) Gas sensing selectivity of the PANI mesh and PANI film for various volatile organic gases. The concentration of gas in all experiments was 10 ppm. The sensitivities for ethanol, 2-propanol, acetone, acetic acid, formaldehyde, toluene, and carbon dioxide are multiplied by 10. (b) Ammonia gas sensing performance under different concentration of interfering gas species, including formaldehyde, toluene, and carbon dioxide ranging from 10 ppb to 50 ppb. The concentration of ammonia gas was 20 ppb.

The selectivity among various gases is an essential parameter for any gas sensors. As displayed in Fig. 6a, the PANI mesh exhibited a specific response to ammonia gas over other volatile organic compounds. The gas sensing sensitivity of the PANI mesh to 10 ppm ammonia gas was 136, 165, 497, 79, 137, 262, and 205 times higher than that to 10 ppm ethanol, isopropanol, acetone, acetic acid, formaldehyde, toluene, and carbon dioxide, respectively. The PANI mesh also exhibited a slightly higher selectivity to ammonia than the PANI film did. Another important parameter of a gas sensor is the stability under interfering gases. Fig. 6b shows the gas sensing stability of the PANI mesh to 20 ppb ammonia gas with the presence of three other typically existing pollutant gases, formaldehyde, toluene, and carbon dioxide with range of 10 ppb to 50 ppb, respectively. The variation of the sensitivity for all three gases were within 15%, which further confirms the excellent selectivity and stability of PANI mesh ammonia sensor.

To examine the repeatability of the PANI mesh in high-performance ammonia gas sensing, the PANI mesh was exposed to 10 ppm ammonia gas in four cycles, and no obvious decline in the sensing sensitivity was observed (Fig. S8, ESI†). The sensing performances of the PANI meshes generated from various polymerization times were also experimentally investigated, and the sample polymerized for 20 min exhibited the highest sensitivity (Fig. S9, ESI†), which could be attributed to its higher proportion of emeraldine in the deposited PANI and the larger specific surface area than the other samples. For the sample produced with a shorter polymerization time, the deposited PANI was not adequate to form a complete mesh with full coverage in the trench. For the sample produced with a longer polymerization time, the overoxidation of the emeraldine form of PANI and the further growth of the PANI grains led to a decrease in the sensitivity of the mesh (Fig. S10, ESI†). The stability of the PANI mesh in high-performance ammonia gas sensing under various environment humidity was also examined by exposing to 20 ppb ammonia gas, while the environmental relative humidity was maintained at 40%, 60%, and 80%, respectively, and the differences in the sensing sensitivity were less than 10% during the 15 min experiments, which is consistent with previous study^36^ (Fig. S11, ESI†). However, when placed in the environment with high humidity for longer time, the sensitivity of the gas sensor may suffer further from the gradually increasing conductivity of the HCl doped PANI mesh.^37^

**Fig. 7:**
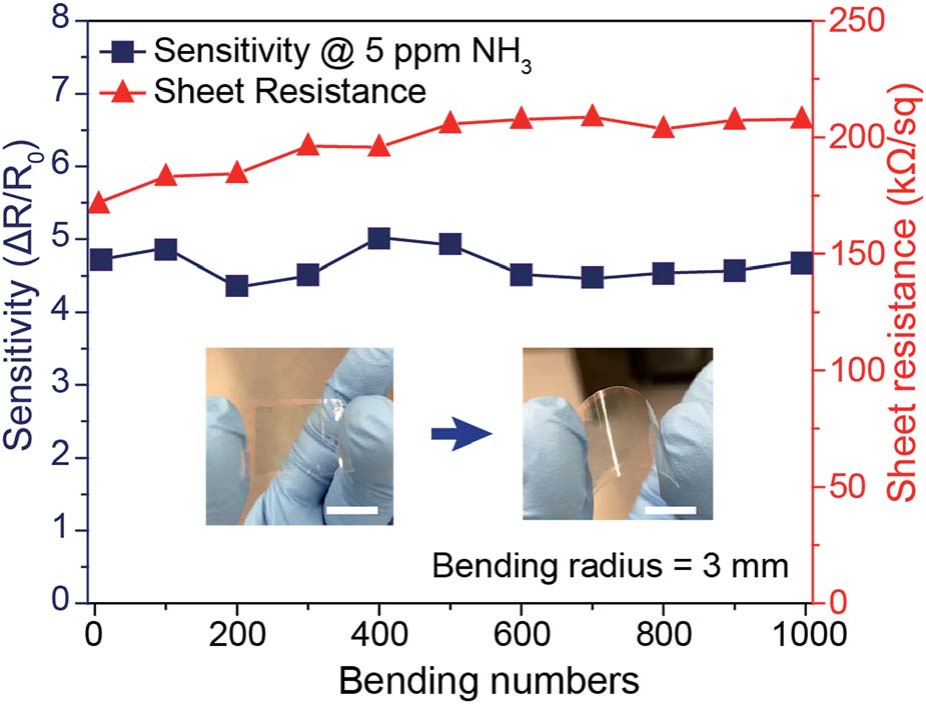
Mechanical stability of the PANI ammonia gas sensor prototype. Variations in the sheet resistance (red curve) and sensing sensitivity (blue curve) to 5 ppm ammonia gas *versus* the number of cycles of bending (tensile loading) to a radius of 3 mm. The scale bars represent 1 cm.

In addition to the enhanced sensing performance of the PANI mesh, the embedded nature of the PANI micromesh mitigated the risk of delamination from the substrate and enhanced its stability under mechanical bending. Fig. 7 provides the results of mechanical stability tests performed on the PANI ammonia gas sensor prototype under cyclic bending stress. The variation in the sheet resistance as a function of the number of cycles of tensile bending to a radius of 3 mm clearly indicates that the sheet resistance remained within 25% of its original value (in a range from 171.3 kΩ sq^−1^ to 207.9 kΩ sq^−1^). Meanwhile, the sensing sensitivity to 5 ppm ammonia gas varied by only 14.4% over 1000 bending cycles. This remarkable stability of the PANI micromesh makes such devices promising candidates for use in flexible and wearable applications.

## Conclusion

In summary, we have demonstrated a new type of high-performance electronic ammonia gas sensor with superior flexibility and excellent optical transmittance. The sensor was fabricated *via in situ* polymerization of aniline monomer on a catalytic sacrificial Cu micromesh template embedded in a flexible substrate prepared by solution-processed microfabrication. The hierarchical nanostructured PANI mesh increased the specific surface area relative to the PANI film, thus enhancing the sensitivity and SNR of the gas sensor. The assembled sensor exhibited high-performance sensing of ammonia gas with concentrations ranging from 2.5 ppb to 100 ppm and an excellent near-neutral color transparency (88.4% at 600 nm wavelength). The embedded nature of the structure also improved the mechanical stability of the sensor under peeling and bending stress, and no obvious decrease in the sensing performance was observed after 1000 bending cycles. The synthetic approach can be easily adapted to prepare flexible and even stretchable sensors. With the superior performance and potentially low-cost, high-throughput fabrication process, our transparent PANI ammonia gas sensor holds the promise for broad applications in handheld or wearable flexible electronic or optoelectronic devices.

## Conflicts of interest

There are no conflicts to declare.

## Supplementary Material

RA-008-C7RA13516E-s001
