# Supplementary material for: Highly transparent and flexible polyaniline mesh sensor for chemiresistive sensing of ammonia gas
Source: RSC Adv. 2018 Jan 30;8(10):5312–20. doi: 10.1039/c7ra13516e (PMC9078194; doi:10.1039/c7ra13516e)
Supplement: RA-008-C7RA13516E-s001 [file RA-008-C7RA13516E-s001.pdf]

Electronic Supplementary Material (ESI) for RSC Advances.
This journal is © The Royal Society of Chemistry 2018

Electronic Supplementary Information for

Highly Transparent and Flexible Polyaniline Mesh Sensor for Chemiresistive Sensing of Ammonia Gas

Jingxuan Cai[a,b], Cuiping Zhang[a,b], Arshad Khan[a], Chuwei Liang[a,b] and Wen-Di Li[a,b]

[a]*Department of Mechanical Engineering, The University of Hong Kong, Pokfulam, Hong Kong, China*

[b]*HKU-Zhejiang Institute of Research and Innovation (HKU-ZIRI), Hangzhou 311300, Zhejiang, China.*

Contact e-mail: liwd@hku.hk

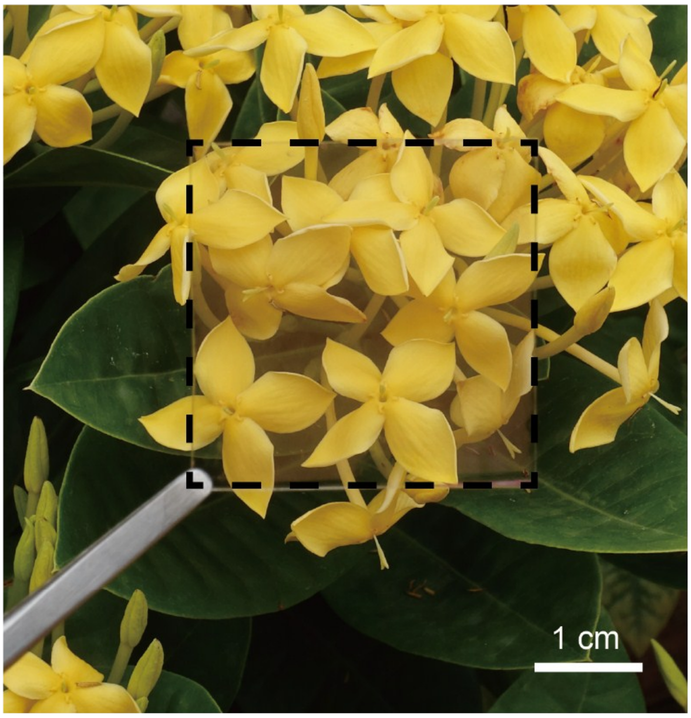

Figure S1. Photograph of the original Cu mesh on a cyclic olefin copolymer (COC) film; the sheet resistance and transparency of the Cu mesh were 0.3 Ω/□ and 72%, respectively.

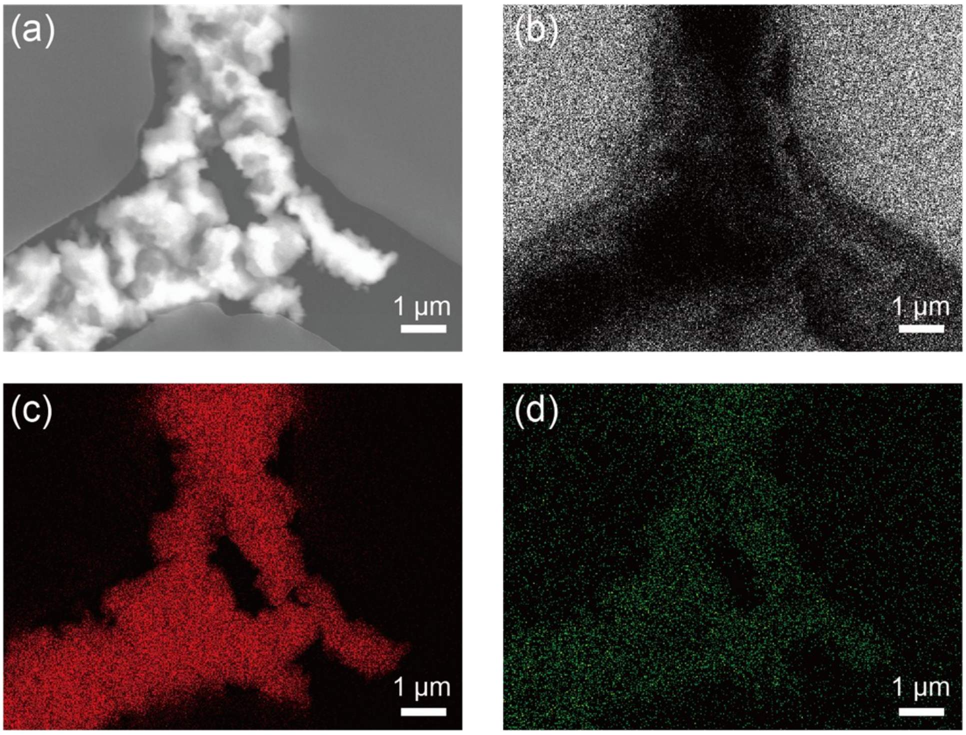

Figure S2. SEM-EDS analysis of a partially etched Cu mesh after 3 min in the aniline polymerization solution on COC film. (a) SEM image; elemental mapping: (b) carbon, (c) copper, and (d) chlorine.

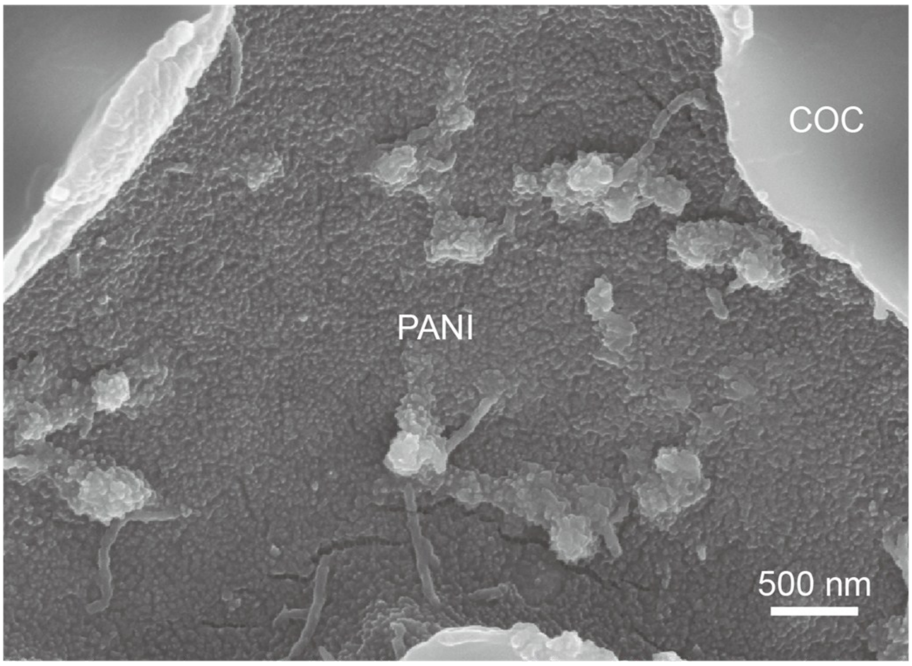

Figure S3. SEM micrograph of the PANI nanostructures on the PANI mesh after 20 min in the aniline polymerization solution.

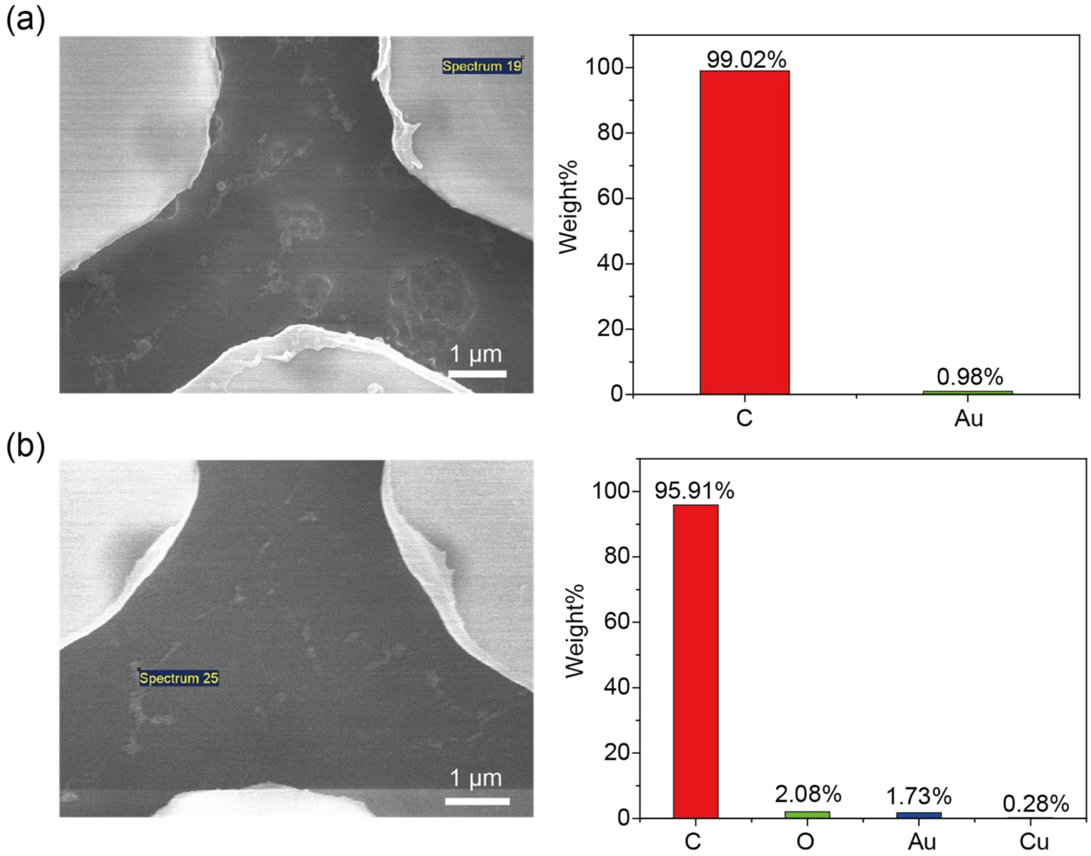

Figure S4. SEM-EDS analysis of a PANI mesh after 20 min in the aniline polymerization solution on COC film. (a) Analysis on the uncovered COC area, and (b) on PANI mesh.

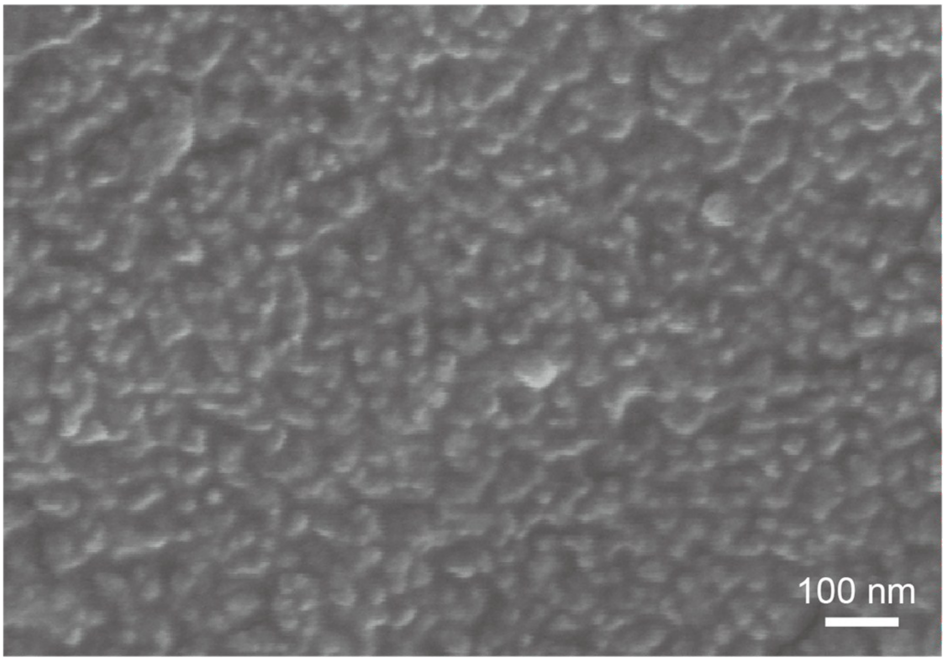

Figure S5. Zoom-in SEM micrograph of the PANI nanoparticles on the PANI mesh after 20 min in the aniline polymerization solution.

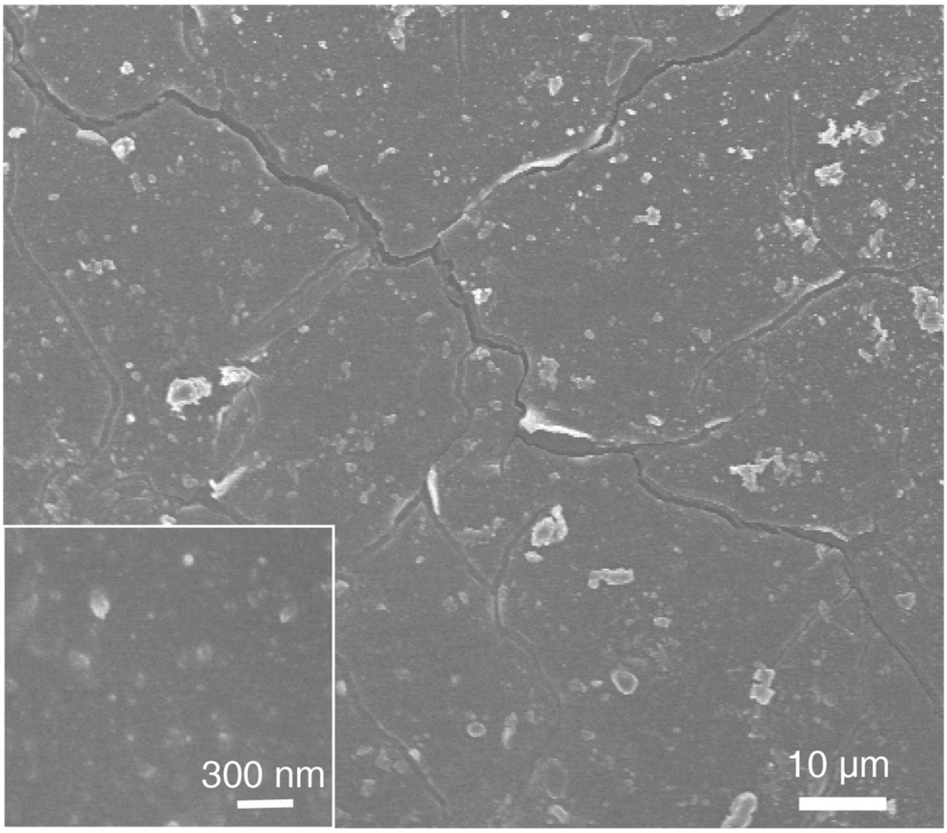

Figure S6. SEM micrograph of a continuous PANI film fabricated by immersing an unmodified COC film in the aniline polymerizing solution for 20 min. (inset) Zoom-in SEM micrograph showing the morphology on the continuous PANI film.

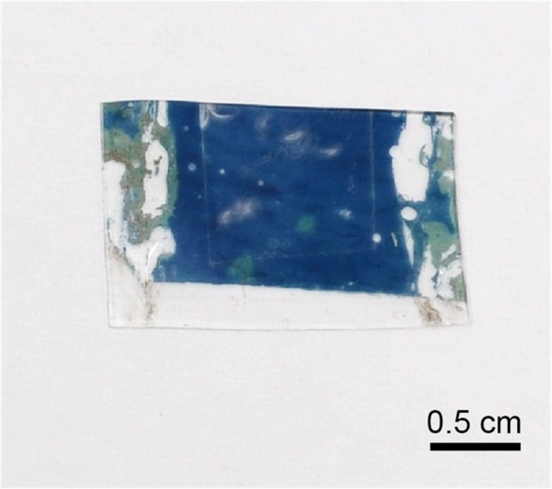

Figure S7. Photograph of a continuous PANI film fabricated by immersing a Cu-coated COC film in the aniline polymerizing solution for 30 min. The Cu-coated COC film was fabricated by immersing a palladium-activated COC film in a Cu electroless plating bath at 40 ℃ for 10 min.

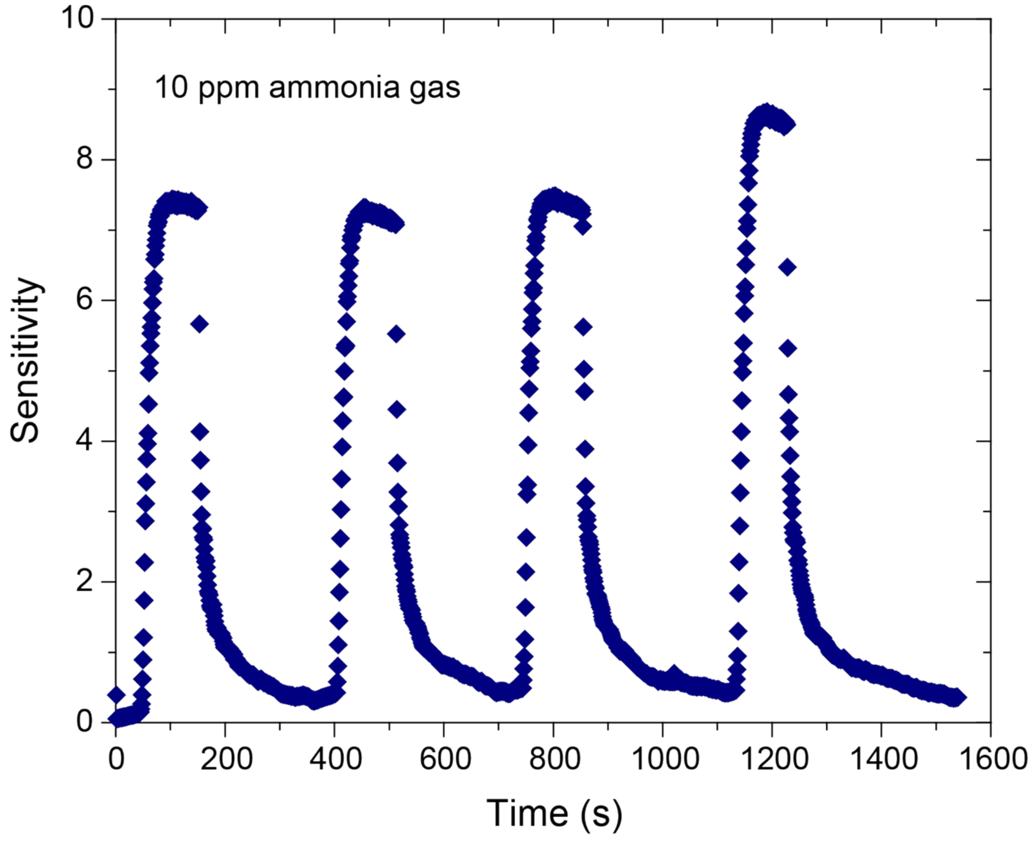

Figure S8. Sensing performance of the PANI mesh under exposure to ammonia gas of 10 ppm concentration for four consecutive cycles. The PANI mesh was generated from 20-min polymerization.

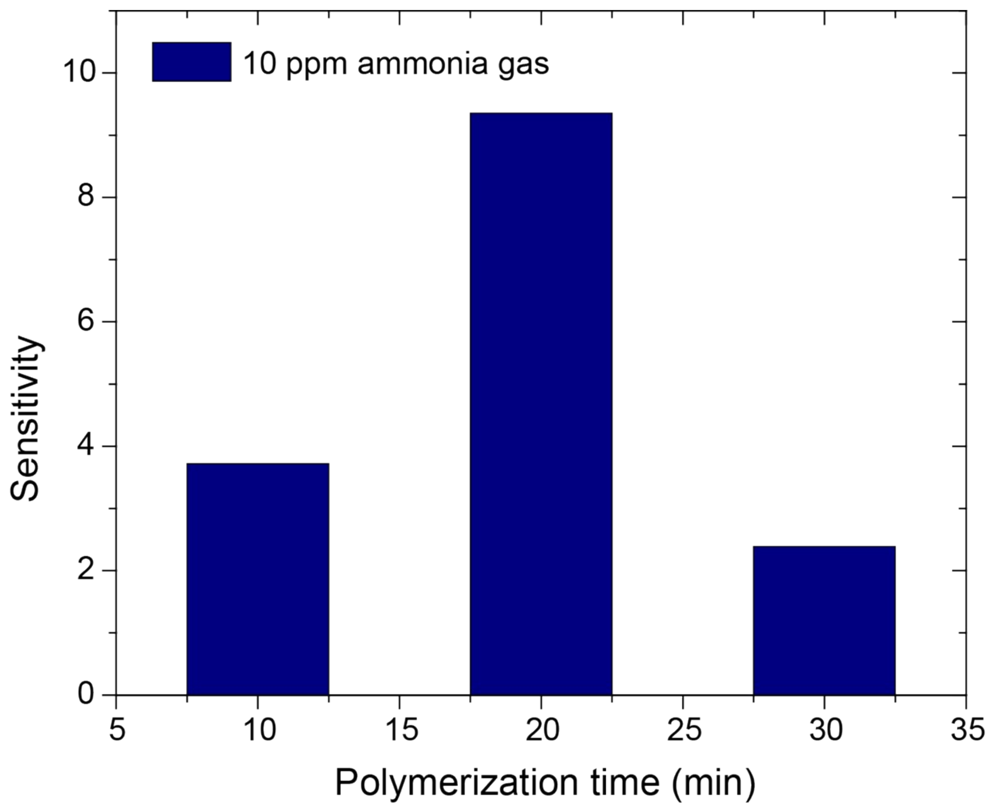

Figure S9. Sensing performance of the PANI mesh generated from various lengths of polymerization time under exposure to ammonia gas of 10 ppm.

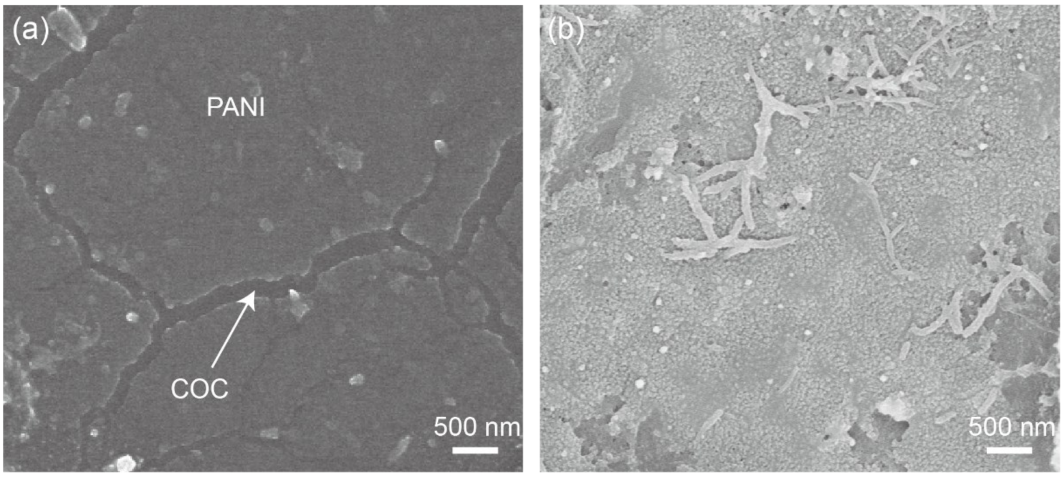

Figure S10. SEM micrographs of the PANI nanostructures on the PANI mesh after (a) 10 min, and (b) 30 min in the aniline polymerization solution.

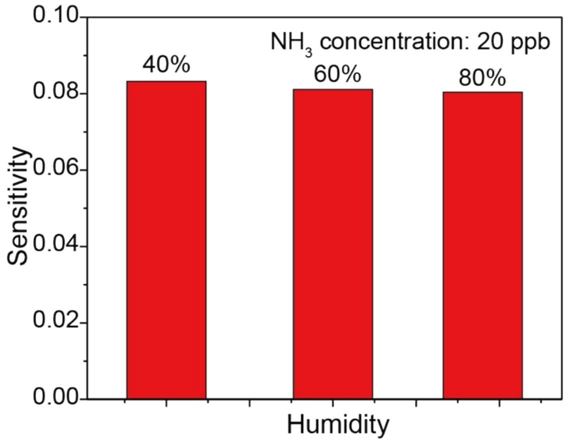

Figure S11. Sensing performance of the PANI mesh under exposure to ammonia gas of 20 ppb at different environmental relative humidity. The uncertainty of humidity was 5%.
